# Study on the Hepatotoxicity of Emodin and Its Application in the Treatment of Liver Fibrosis

**DOI:** 10.3390/molecules29215122

**Published:** 2024-10-30

**Authors:** Yurou Guo, Jiawen Song, Yushi Liu, Minghao Yuan, Wenxiao Zhong, Yiping Guo, Li Guo

**Affiliations:** 1 State Key Laboratory of Southwestern Chinese Medicine Resources, Chengdu University of Traditional Chinese Medicine, Chengdu 611137, China; guoyurou@stu.cdutcm.edu.cn (Y.G.); songjiawen@stu.cdutcm.edu.cn (J.S.); liuyushi@stu.cdutcm.edu.cn (Y.L.); yuanminghao@stu.cdutcm.edu.cn (M.Y.); zhongwenxiao@stu.cdutcm.edu.cn (W.Z.); 2 School of Pharmacy, Chengdu University of Traditional Chinese Medicine, Chengdu 611137, China

**Keywords:** emodin, liver fibrosis, toxicity, tetrandrine, co-delivery system

## Abstract

Emodin (EMO) is an anthraquinone compound derived from *Rheum palmatum* L., which has rich pharmacological activity. However, studies have shown that EMO may cause hepatotoxicity. In this study, EMO was combined with tetrandrine and prepared as lipid nanoparticles (E-T/LNPs). The anti-liver fibrosis activity of EMO before and after formulation was evaluated by zebrafish and mice. In addition, the toxicity of EMO and E-T/LNPs was compared and the toxicity–efficacy concentrations of E-T/LNPs in zebrafish were verified. E-T/LNPs are morphologically stable (particle size within 100 nm), have high encapsulation efficiency and good stability, and are capable of long-lasting slow release in vitro. The combination and preparation can reduce the toxicity and enhance the effect of EMO, and increase the toxicity and effect concentration of E-T/LNPs in vivo. In a short period, low doses of E-T/LNPs can be used for the treatment of liver fibrosis; high doses of E-T/LNPs cause toxicity in vivo. Immunohistochemistry showed that E-T/LNPs inhibited hepatic fibrosis by downregulating the levels of IL-1β and TGF-β. Based on the advantages of combination therapy and nanotechnology, it can play a role in reducing the toxicity and increasing the efficacy of EMO in the treatment of liver fibrosis.

## 1. Introduction

Liver fibrosis is a reversible pathological change caused by chronic liver injury and it is a necessary developmental stage of cirrhosis and hepatocarcinoma [1,2,3]. As a result of impaired liver function, hepatic stellate cells (HSCs) are activated by inflammatory factors and transformed into a myofibroblast phenotype, ultimately leading to liver fibrosis [4]. Therefore, it is necessary to eliminate liver inflammation to inhibit fibrosis. However, due to the complexity of liver fibrosis causes, treatment options are limited, and there is no ideal clinical therapeutic agent yet. Traditional Chinese medicine (TCM) has a long history of application in hepatoprotection and has been effective in preventing and controlling liver fibrosis caused by various etiologies [5]. Therefore, TCM has a good prospect of application in treating hepatic fibrosis.

Emodin (EMO), a natural anthraquinone compound derived from *Rheum palmatum* L., has good pharmacological activities [6]. It is widely used in treating various stages of liver disease, including hepatitis, fatty liver disease, liver fibrosis, cirrhosis, and hepatocellular carcinoma [7,8,9]. However, recent studies have shown that long-term use of high doses of EMO may lead to hepatotoxicity, nephrotoxicity, and reproductive toxicity [10,11,12,13]. Additionally, its poor water solubility and low bioavailability limit its clinical application. Therefore, there is an urgent need for a strategy to enhance solubility, reduce toxicity, and improve its efficacy in treating liver fibrosis.

Combination therapy refers to the simultaneous administration of two or more drugs, which not only enhances the efficacy of the drugs but also reduces their toxicity and has a variety of advantages [14,15]. Tetrandrine (TET) is an alkaloid with excellent anti-inflammatory activity, which can treat liver disease by inhibiting the stimulation of the liver by inflammatory factors [16,17]. Based on the advantages of the combination, we chose the combination of EMO and TET with nanotechnology to increase the efficacy, reduce the toxicity, and improve the bioavailability in treating liver fibrosis [18,19,20,21,22,23].

In this study, we constructed, for the first time, a lipid nano-delivery system (E-T/LNPs) co-loaded with EMO and TET for the treatment of liver fibrosis. Firstly, E-T/LNPs were characterized and quality-assessed. Subsequently, we explored the toxicity–efficacy concentrations of E-T/LNPs and examined whether EMO alleviated the toxicity through combination and formulation in zebrafish. In addition, the anti-hepatic fibrosis activity of EMO was evaluated before and after combination and formulation by zebrafish and mice.

## 2. Results

### 2.1. Determination of Physical and Chemical Properties

**Figure 1:**
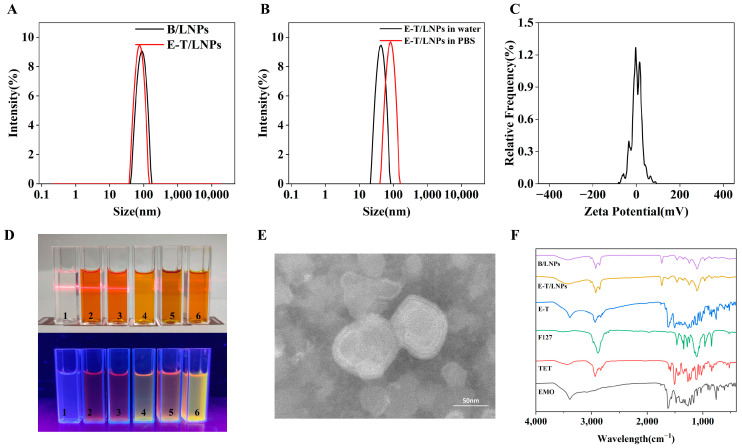
Characterization of E-T/LNPs. (**A**) Particle size distribution of B/LNPs and E-T/LNPs. (**B**) Particle size distribution of E-T/LNPs in different media. (**C**) Zeta potential of E-T/LNPs. (**D**) Tyndall effect of E-T/LNPs in natural light and fluorescence under 302 nm irradiation. (1: B/LNPs in water. 2–5: E-T/LNPs in water, PBS, ethanol, DMSO. 6: TET and EMO in ethanol). (**E**) TEM of E-T/LNPs. (**F**) The FT-IR spectra (B/LNPs, E-T/LNPs, E-T, F127, TET, EMO). The characteristic signal peaks of both EMO and TET disappeared in the E-T/LNPs group.

Particle size and zeta potential: The method for optimizing the process of E-T/LNPs is described in the Supplementary Materials (Tables S1–S3 and Figure S1), followed by the characterization of the preferred E-T/LNPs. The results showed that the particle sizes of blank lipid nanoparticles (B/LNPs) and E-T/LNPs in water were 88.09 ± 0.15 nm and 77.80 ± 1.01 nm, respectively (Figure 1A), suggesting that the particle sizes of E-T/LNPs did not obviously increase after drug loading. According to Figure 1B, the particle sizes of E-T/LNPs in water and phosphate-buffered solution (PBS) were 77.80 ± 1.01 nm and 85.11 ± 1.02 nm, respectively. The zeta potential of E-T/LNPs (Figure 1C) was −6.77 ± 3.08 mV.

Tyndall effect and fluorescence: The results (Figure 1D) showed that E-T/LNPs exhibited the Tyndall effect in PBS and water, but not in ethanol and dimethyl sulfoxide (DMSO). Similarly, when placed in a fluorescent environment, E-T/LNPs exhibited no fluorescence in water or PBS, but emitted fluorescence of varying intensities in organic reagents. The detection results of both initially indicated that the preparation of E-T/LNPs was successful. E-T/LNPs were non-fluorescent in water or PBS and emitted fluorescence of varying intensity in organic reagents, because the drug was encapsulated in the middle of the phospholipid bilayer and the EMO fluorescence was masked; when in an organic system, the drug was released to varying extents, and fluorescent effects could be detected.

Transmission electron microscope (TEM): As shown in Figure 1E, it could be observed that the outer layer of E-T/LNPs consisted of a phospholipid bilayer and formed round-like structures with a diameter within 100 nm.

Fourier-transform infrared spectroscopy (FT-IR)**:** As shown in Figure 1F, in the absorption pattern of EMO, 873 cm^−1^, 761 cm^−1,^ and 727 cm^−1^ were the vibrational peaks of C–C and C–H bonds on the benzene ring backbone, and the stretching vibration of the C–O bond was at 1334 cm^−1^, 1264 cm^−1^ and 1095 cm^−1^ [24]. The sharp peak at 1620 cm^−1^ was the vibrational peak of C=O, and the broad peak at 3394 cm^−1^ was the absorption peak of O–H in EMO [25]. The FT-IR spectra of TET showed stretching vibration and out-of-plane vibration of the C–H bond at 2933 cm^−1^ and 840 cm^−1^, respectively. Absorption peaks at 1606 cm^−1^, 1578 cm^−1^, and 1508 cm^−1^ were attributed to the benzene ring and the asymmetric stretching vibration of C–O–C at 1024 cm^−1^ [26]. The FT-IR pattern of poloxamer 407 (F127) was dominated by three absorption peaks at 2898 cm^−1^, 1344 cm^−1^, and 1110 cm^−1^, which were attributed to C–H, O–H, and C–O bonds, respectively. According to the FT-IR pattern of E-T (the fixture of EMO and TET), the characteristic absorption peaks in both EMO and TET were detected, indicating that their physical mixtures do not bond covalently, but exist separately as monomers. In the FT-IR spectra of both B/LNPs and E-T/LNPs, there were only five main peaks at 2929 cm^−1^, 1739 cm^−1^, 1467 cm^−1^, 1247 cm^−1^, and 1110 cm^−1^. The signal peaks before and after drug loading were the same, no new signals appeared after drug loading, and all the characteristic signals of EMO and TET disappeared, indicating that the preparation of E-T/LNPs was successful.

Stability: As shown in Table S4, with the passage of time, the particle size of E-T/LNPs increased by about 15 nm, the encapsulation efficiency (EE) of EMO and TET decreased by 17.11% and 23.74%, and the drug loading (DL) decreased by 0.88% and 1.21%, respectively. These data indicated that the stability of E-T/LNPs was good.

Drug release in vitro: As shown in Figure S2, pure EMO and TET demonstrated significant differences in the release process between the monomer drug group and the E-T/LNPs group. In the monomer drug group, cumulative release (CR) gradually stabilized at 8 h and reached complete stability within 24 h. In the E-T/LNPs group, the drug had been released slowly and continuously and did not reach a plateau in 24 h. In addition, the CR of EMO and TET were 26.62% and 25.77% in the monomer drug group, whereas the CR was significantly increased in the E-T/LNPs, with the CR of EMO and TET being 53.06% and 55.00%, respectively. The results show that E-T/LNPs could greatly improve the solubility of the EMO and TET. Moreover, the E-T/LNPs were capable of sustained drug release, which was more conducive to the prolonged retention and sustained action of the drugs in vivo.

### 2.2. Toxicity–Efficacy of E-T/LNPs

**Figure 2:**
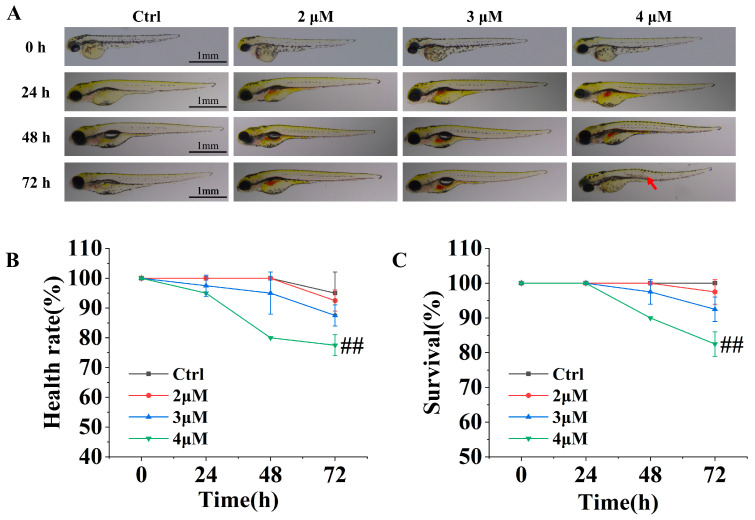
The zebrafish after co-incubation in different concentrations of drugs for different periods. (**A**) Morphology of zebrafish (red arrows point to abnormal zebrafish morphology). (**B**) Survival of zebrafish. (**C**) Health rate of zebrafish. ^#^, Compared with Ctrl group, ^##^ *p* < 0.01.

Zebrafish (Danio rerio), a tropical aquatic animal, is an ideal model for pharmacology and toxicology due to its high genetic homology with human genes, ease of culture, short reproduction cycle, high spawning capacity, and ease of observation [27,28,29]. As shown in Figure 2, at a concentration of 2 μM of E-T/LNPs, the morphology of zebrafish remained normal and well developed, with the results not being significantly different from those of the Ctrl group (*p* > 0.05). When the concentration of E-T/LNPs reached 3 μM, zebrafish larvae began to show decreased viability and some deaths occurred (*p* < 0.05). In the high-concentration group (4 μM), zebrafish larvae exhibited significant deformities and mortality at 48–72 h as the drug was gradually absorbed (*p* < 0.01). The results indicate that E-T/LNPs are safe and non-toxic to zebrafish at a low concentration of 2 μM, but cause toxicity when the concentration exceeds 4 μM. Therefore, in subsequent pharmacological experiments, a safe concentration within 2 μM will be selected for the treatment of zebrafish larvae.

### 2.3. E-T/LNPs Reduced the Toxicity of EMO

**Figure 3:**
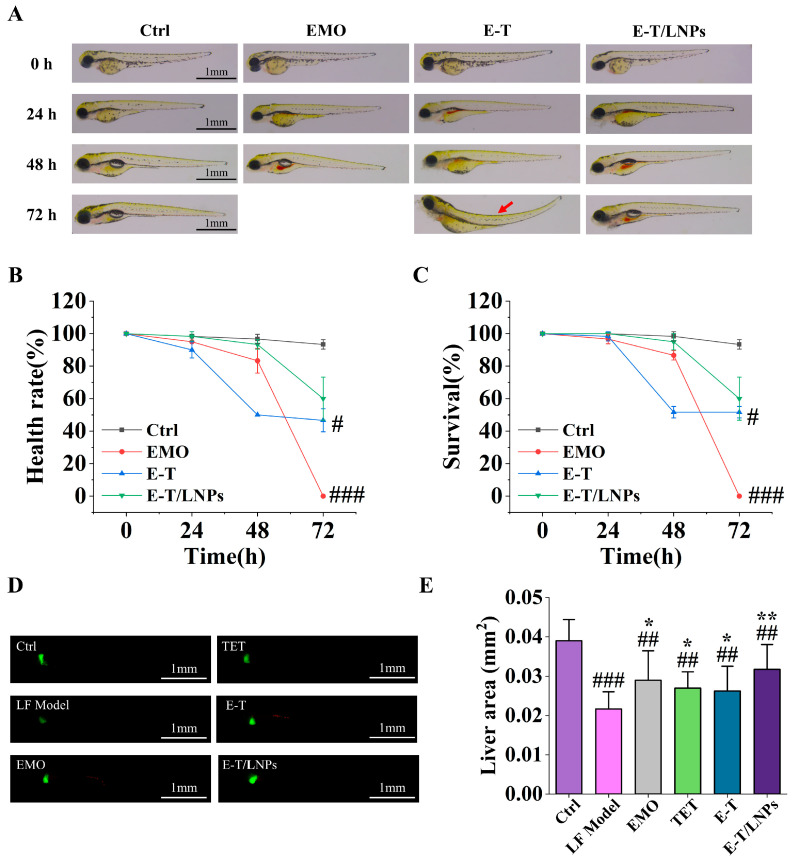
The zebrafish after co-incubation with different drugs. (**A**) Morphology of zebrafish (red arrows point to abnormal zebrafish morphology). (**B**) Survival of zebrafish. (**C**) Health rate of zebrafish. (**D**) Liver morphology of zebrafish larvae. (**E**) The liver fluorescence area of zebrafish larvae. ^#^, compared with the Ctrl group, ^#^ *p* < 0.05, ^##^ *p* < 0.01, ^###^ *p* < 0.001. *, compared with the LF Model group, * *p* < 0.05, ** *p* < 0.01.

As shown in Figure 3A–C, the zebrafish in the E-T group exhibited deformities and death in 48 h. At 72 h, the toxicity in the EMO group was more pronounced, including massive mortality, reduced viability, and significant morphological developmental abnormalities (*p* < 0.001). The E-T group did not show more significant changes (*p* < 0.05). Additionally, zebrafish in the E-T/LNPs group remained viable and morphologically normal in 72 h (*p* > 0.05). The results indicate that E-T/LNPs attenuated the toxicity of EMO and improved the safety of E-T/LNPs in vivo.

### 2.4. Therapeutic Effect of E-T/LNPs on Liver Fibrosis in Zebrafish

As shown in Figure 3D–E, the fluorescent areas represent the liver tissue of zebrafish larvae. The larvae in the control (Ctrl) group displayed clear and strong fluorescent signals. However, zebrafish in the LF (liver fibrosis) Model group showed significantly weaker fluorescent signals and a substantial reduction in liver area (*p* < 0.001). The zebrafish liver area was increased in all administration groups compared with the LF Model group (*p* < 0.05) and the fluorescence signal was enhanced. The change in the liver area of the E-T/LNPs group was particularly significant (*p* < 0.01). The results demonstrate that E-T/LNPs exhibited excellent anti-hepatic fibrosis ability in zebrafish.

### 2.5. Masson Staining and Liver Biochemical Index

**Figure 4:**
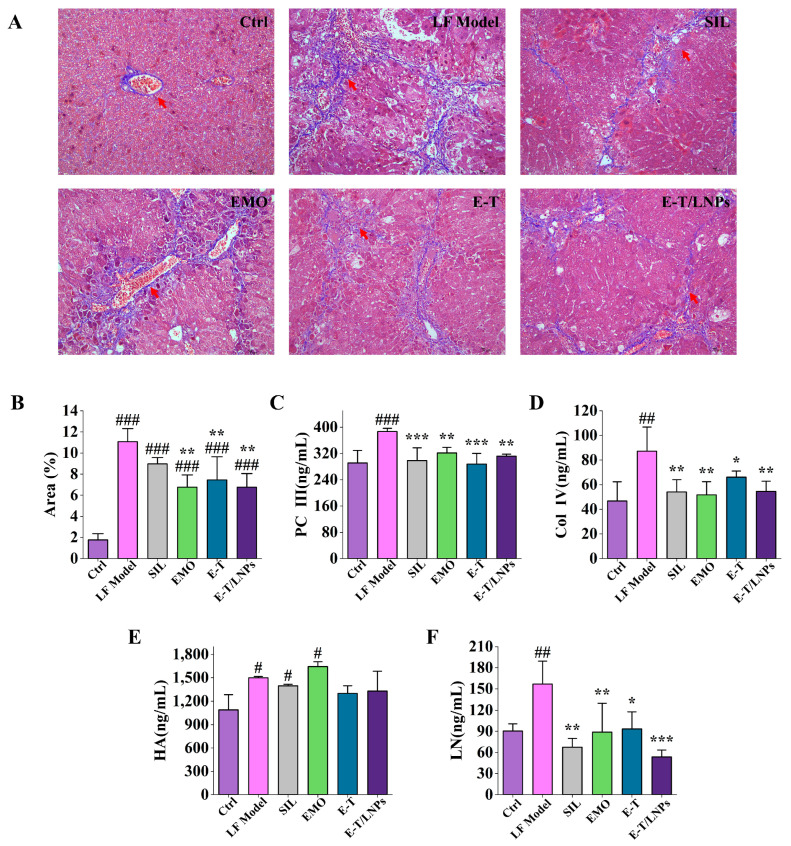
(**A**) Masson-stained sections of the liver of mice (400×). Red arrow: collagen fibers. (**B**) Masson-positive expression area. (**C**) Liver PC III vitality. (**D**) Liver Col IV vitality. (**E**) Liver HA vitality. (**F**) Liver LN vitality. ^#^, compared with the Ctrl group, ^#^ *p* < 0.05, ^##^ *p* < 0.01, ^###^ *p* < 0.001. *, compared with the LF Model group, * *p* < 0.05, ** *p* < 0.01, *** *p* < 0.001.

The results of stained sections of liver tissue from each group of mice with quantification are shown in Figure 4A,B. Compared with the Ctrl group, a large number of blue collagen fibers were aggregated in the LF Model group (*p* < 0.001), indicating that CCl_4_ successfully induces liver fibrosis in mice. Compared with the LF Model group, there was a decrease in collagen fibers in the liver tissue of the EMO, E-T, and E-T/LNPs groups (*p* < 0.01), suggesting that the drug was effective in reducing the collagen fiber deposition after administration. Compared with the silymarin (SIL) group, there was no significant difference in the EMO, E-T, and E-T/LNPs groups (*p* > 0.05). The positive expression area in the E-T/LNPs group was diminished compared with that in the E-T group, indicating that the anti-liver fibrosis effect of E-T/LNPs was enhanced.

As shown in Figure 4C–F, the levels of type III procollagen (PC III), type IV collagen (Col IV), laminin (LN) and hyaluronic acid (HA) were significantly higher in the LF Model group compared with the Ctrl group (*p* < 0.05). And all levels in the treatment group were reduced to varying degrees (*p* < 0.01). There was no significant difference in the overall levels in the E-T/LNPs group compared with the SIL group, except that LN levels were lower than in the SIL group (*p* > 0.05). Both Col IV and LN levels were reduced in the E-T/LNPs group compared with the E-T group. E-T/LNPs not only improve the efficacy of the EMO, but also significantly reduce the level of the E-T/LNPs group compared with the E-T group. These results suggest that 20 mg/kg of E-T/LNPs could restore the physiological function of the liver and alleviate the CCl_4_-induced liver fibrosis.

### 2.6. HE Staining and Serum Biochemical Index

**Figure 5:**
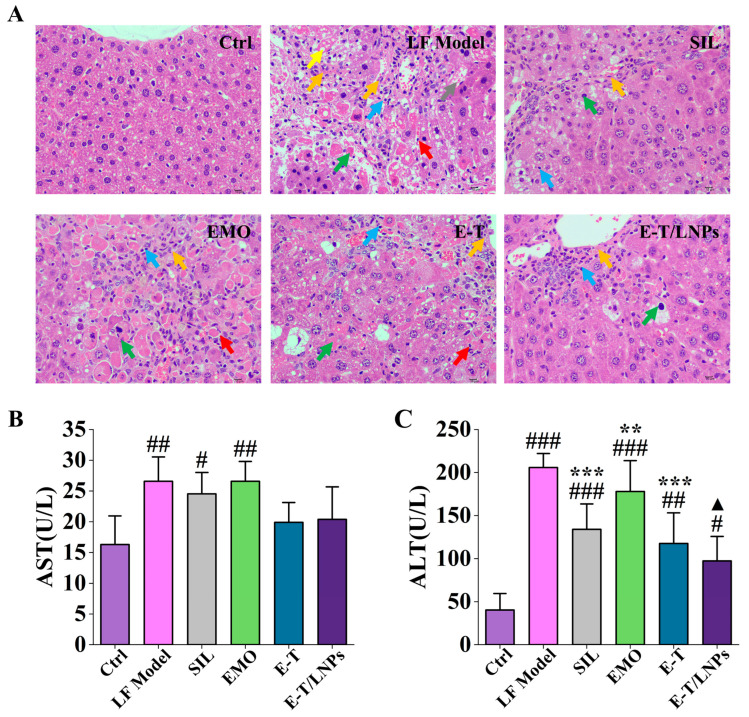
(**A**) HE-stained sections of the liver of mice (yellow: fatty degeneration; green: cellular necrosis; orange: fibrous tissue proliferation; red: neutrophils; blue: lymphocytes; gray: hepatocyte ballooning) (400×). (**B**) Serum AST vitality. (**C**) Serum ALT vitality. ^#^, compared with the Ctrl group, ^#^ *p* < 0.05, ^##^ *p* < 0.01, ^###^ *p* < 0.001. *, compared with the LF Model group, ** *p* < 0.01, *** *p* < 0.001. ^▲^, compared with the Ctrl group, ^▲^ *p* < 0.05.

According to the results shown in Figure 5A, the liver tissue of the Ctrl group appeared structurally intact with morphologically normal and neatly arranged hepatocytes, and no obvious lesions were observed. After 8 weeks of CCl_4_ administration, a large number of hepatocytes were degenerated and necrotic, the central vein was dilated with extensive fibrous tissue proliferation, and pseudolobules had formed. After 3 weeks, the treatment groups showed varying degrees of improvement. Specifically, the EMO group exhibited some improvement but still showed pronounced necrosis of liver tissue and obvious bundles of fibroblasts with inflammatory infiltration. The E-T group showed that some hepatocytes were degenerated or necrotic, the cellular morphology and structure were more blurred, and there was a small amount of fibrous tissue generation, which was relatively reduced compared with the LF Model group. The improvement was particularly evident in the SIL group, which demonstrated regeneration of liver tissue structure, reduction in inflammatory infiltration, and a very small amount of fibroblastogenesis. The E-T/LNPs group likewise showed a reduction in the extent of the lesions, with decreased fibrous tissue proliferation and hepatocyte regeneration, comparable to that seen in the SIL group. These results suggested that 20 mg/kg of E-T/LNPs was able to attenuate CCl_4_-induced liver damage and the reduction in inflammation.

As shown in Figure 5B,C, aspartate aminotransferase (AST) and alanine aminotransferase (ALT) levels were significantly higher in the LF Model group compared with the Ctrl group (*p* < 0.01). There was no significant difference in AST levels in the E-T/LNPs group compared with the Ctrl group (*p* > 0.05). The AST and ALT levels were reduced in mice receiving treatment compared with the LF Model group (*p* < 0.05), while the ALT levels decreased in the following order: EMO, E-T, and E-T/LNPs groups. These results indicate that the EMO improved the inflammatory response in mice, and the therapeutic effect was enhanced after combination and formulation, consistent with the results from HE-stained sections.

### 2.7. E-T/LNPs Reduced TGF-β and IL-1β Levels

**Figure 6:**
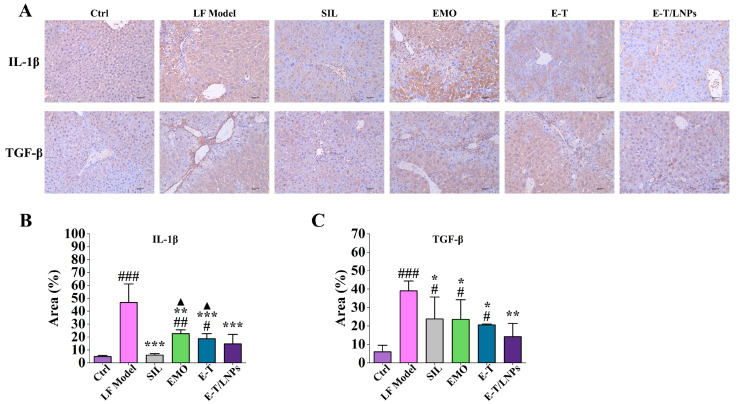
Effect of E-T/LNPs on the expression of IL-1β and TGF-β. (**A**) Immunohistochemistry staining of IL-1β and TGF-β in the liver tissues (100×). (**B**) Quantitative results of IL-1β in the liver tissues. (**C**) Quantitative results of TGF-β in the liver tissues. ^#^, compared with the Ctrl group, ^#^ *p* < 0.05, ^##^ *p* < 0.01, ^###^ *p* < 0.001. *, compared with the LF Model group, * *p* < 0.05, ** *p* < 0.01, *** *p* < 0.001. ^▲^, compared with the SIL group, ^▲^ *p* < 0.05.

As shown in Figure 6, the levels of interleukin-1β (IL-1β) and transforming growth factor (TGF-β) were significantly upregulated in the LF Model group after CCl_4_ treatment (*p* < 0.001). After drug intervention, the expression was significantly downregulated in all administered groups (*p* < 0.05). The downregulation of IL-1β was most significant in SIL and E-T/LNPs, which was not significantly different from the Ctrl group (*p* > 0.05). And the levels of IL-1β and TGF-β in the EMO group, E-T group and E-T/LNPs group showed a sequential downward trend. In addition, there was no significant difference in the levels of IL-1β and TGF-β between the SIL group and the E-T/LNPs group (*p* > 0.05.).

## 3. Discussion

Nano-delivery systems are a novel form of drug delivery that has been studied in recent years, and they have been widely used in treating a variety of diseases due to their multiple advantages, including improving drug solubility, increasing biocompatibility, prolonging circulation time of drugs in the body, and reducing toxicity and side effects [30]. In this study, we developed a nano-drug co-delivery system E-T/LNPs containing EMO and TET and characterized its morphology, particle size, zeta potential, EE, DL and stability. It was observed that the particle size of E-T/LNPs in PBS is larger than that in water, which may be due to the ions in the PBS affecting the aggregation behavior of the E-T/LNPs, resulting in a change in particle size [31]. This study found that nanomedicine with a diameter of less than 200 nm is ideal for oral administration, indicating that E-T/LNPs can be effectively absorbed and transported in vivo [32]. The particle size of E-T/LNPs was 77.80 ± 1.01 nm, which is a suitable particle size to be effectively absorbed and transported in vivo. The nanoparticles with negative zeta potentials have higher kinetic stability than those with positive zeta potentials [33], whereas E-T/LNPs exhibit negative zeta potentials, suggesting that E-T/LNPs have good stability. Since EMO has fluorescence [34], it can emit fluorescence under light irradiation at 302 nm. E-T/LNPs were non-fluorescent in water and PBS, and emitted fluorescence of varying intensity in organic reagents because the drug was encapsulated in the middle of the phospholipid bilayer and the EMO fluorescence was masked; when in an organic system, the drug was released to varying extents, and fluorescent effects could be detected. And similar results were seen in the Tyndall effect of E-T/LNPs, suggesting that EMO and TET can be encapsulated in the form of lipid nanoparticles. In addition, the stability test and in vitro release test also showed that E-T/LNPs had good stability and could be released slowly.

EMO has attracted significant attention due to potential hepatotoxicity [12]. It has been found that EMO can exhibit hepatotoxicity by disrupting hepatic antioxidant homeostasis, particularly through glutathione and xanthine metabolism [13]. Studies have shown that administering toxic drugs in combination with other therapies or in the form of nanoparticles can inhibit their excessive accumulation in the body, effectively reducing toxicity and improving the therapeutic index [35,36,37,38]. To confirm that EMO can safely and effectively exert antifibrotic effects in vivo, we confirmed the improved toxicity of EMO by comparing the morphology, health rate and survival rate of zebrafish in each group. The results showed that E-T/LNPs were able to reduce deformities and mortality in zebrafish and significantly reduced the toxicity of EMO. And the mechanism of this toxicity reduction may be through the combination with TET and the slow-release effect of the nano-formulations, which reduces the accumulation of the drug in the body and thus improves the safety in vivo.

Studies have shown that low doses of EMO can improve CCl_4_-induced oxidative stress and energy metabolism dysfunction by regulating glucose, lipid, and amino acid metabolism. However, high doses of EMO can lead to liver metabolic disorders and hepatotoxicity [13]. Our study showed that E-T/LNPs did not affect the growth and development of zebrafish in 72 h at a concentration of 2 μM. But the concentration of 4 μM may cause deformities or even death. Thus, the dosage of E-T/LNPs is the key to determining whether it protects the liver or cause liver injury.

Liver fibrosis, resulting from many chronic liver diseases, is caused by continuous hepatocyte damage that activates cells to produce an excessive extracellular matrix, ultimately forming fibrous scars [39]. We validated the anti-liver fibrosis activity of EMO combination before and after formulation using zebrafish and mice. TAA can covalently bind to large molecules in the liver, causing necrosis of liver cells in the central and peripheral regions. Long-term exposure to TAA can activate downstream signaling of TGF–β/Smad3 in damaged liver cells, enabling hepatic stellate cells to acquire a myofibroblast like phenotype, leading to liver fibrosis [40]. The zebrafish experiment results showed that E-T/LNPs can improve the reduction in liver fluorescence area and fluorescence intensity caused by TAA. PC III, Col IV, LN and HA, which are important indicators used to diagnose liver fibrosis in clinical settings, can reflect the degree of hepatocellular damage, liver fibrosis, and the severity of portal hypertension in cirrhosis [41]. Our results indicate that E-T/LNPs improved the histopathology, reduced the production of collagen fibers, decreased the levels of AST, ALT, PC III, Col IV, LN, HA and had a protective effect against CCl_4_-induced liver fibrosis. Additionally, the anti-liver fibrosis activity of E-T/LNPs was significantly better than that of E-T. Since the solubility of EMO and TET in LNPs was significantly increased, it was more favorable for the release and uptake of EMO and TET in vivo, which improved the efficacy. The results demonstrate the excellent antifibrotic activity of E-T/LNPs, consistent with previous reports [17,42]. IL-1β and TGF-β play important roles in the development of hepatic fibrosis. As a pro-inflammatory cytokine, IL-1β can participate in the NLRP3 inflammatory vesicle response and induce inflammation and fibrosis in the liver [43]. Studies have shown that TAA-induced liver fibrosis can be ameliorated by inhibiting IL-1β recruitment and secretion by monocytes/macrophages [44]. In addition, high levels of TGF-β can lead to phenotypic conversion of hepatic stellate cells and hepatocyte death, promoting the development of liver fibrosis and cirrhosis [45]. EMO with TET can reduce liver injury and fibrosis by inhibiting the expression of IL-1β and TGF-β [46,47,48]. Our study showed that E-T/LNPs downregulated the expression of IL-1β and TGF-β, suggesting that the mechanism of E-T/LNPs may be to ameliorate hepatic fibrosis by regulating the expression of inflammatory and fibrogenic factors.

This study confirmed through zebrafish experiments that E-T/LNPs are safe in the short term and determined their optimal antifibrotic concentration. However, liver fibrosis is a chronic liver disease with a prolonged treatment cycle, and we have not yet conducted in-depth research on the long-term toxicity and mechanism of action of E-T/LNPs in vivo. Therefore, future research will focus on further exploring the long-term toxicity and mechanisms of action of E-T/LNPs.

## 4. Materials and Methods

### 4.1. Material

Emodin (98% purity grade) and F127 were purchased from Solarbio Biotechnology Co. Ltd. (Beijing, China). Tetrandrine (98% purity grade) was purchased from Xi’an Xiaocao Plant Development Co. (Xi’an, China). Soy lecithin was purchased from AVT (Shanghai, China) Pharmaceutical Technology Co. Thioacetamide (TAA), phosphoric acid (HPLC grade) and triethylamine (HPLC grade) were purchased from Chengdu Cologne Chemical Co. (Chengdu, China). Methanol (HPLC grade) and acetonitrile (HPLC grade) were purchased from Thermo Fisher Scientific (Shanghai, China). Silymarin (80% purity grade) and CCl_4_ were purchased from Shanghai Rhawn Chemical Technology Co. (Shanghai, China). AST, ALT, PC III, Col IV, LN, and HA kits were purchased from Shanghai MLBIO Biotechnology Co. (Shanghai, China). Ultrapure water was produced by ULUPURE Laboratory Ultrapure Water Machine (Chengdu, China). All other chemicals were of analytical grade.

### 4.2. Animals

Wild-type AB strain and enhanced green fluorescent protein-tagged liver-specific transgenic zebrafish (lfabp: EGFP) were selected as the experimental fish. All zebrafish were cultured at 28 ± 1.0 °C in a 14 h light/10 h dark cycle with culture water at a pH of 7.2–7.5 and a conductivity of 500–550 µS/cm. And their larvae used for the experiments were spawned and hatched in their natural state.

The animals were selected from C57BL/6 male mice (6–8 weeks and weight of 18–22 g) provided by SIBEIFU (Beijing, China) Biotechnology Co. All the mice were housed in a standard animal laboratory (23 ± 2 °C, relative humidity 50 ± 20%) with a 12 h light/12 h dark cycle. The mice had free access to food and clean water ad libitum. All animals were adaptively fed for one week before the experiment. The animal experiment was approved by the Experimental Animal Ethics Committee of Chengdu University of Traditional Chinese Medicine.

### 4.3. Preparation of E-T/LNPs

E-T/LNPs were prepared by ethanol injection [49], and the specific method was adapted as follows. TET and EMO (*m/m* = 1:1) were dissolved in ethanol, to which soy lecithin was added and completely dissolved via ultrasonication as phase A. F127 was added and dissolved in water as phase B. Phase A was then added dropwise to phase B at 750 rpm and 50 °C, with stirring for a determined amount of time, followed by sonication in an ice bath. Finally, high-speed centrifugation was used to remove the insoluble free drug to obtain E-T/LNPs.

### 4.4. Determination of EE and DL

The E-T/LNPs were appropriately diluted with methanol, and the sample was obtained after ultrasonic demulsification. The contents of EMO and TET were determined by the HPLC method [50,51]. The EE and DL were calculated using Equations (1) and (2):

(1) $$EE\left(\%\right)=The\;weight\;of\;drug\;measured/The\;weight\;of\;drug\;added\times 100\%$$

(2) $$DL\left(\%\right)=The\;weight\;of\;drug\;measured/The\;weight\;of\;all\;material\times 100\%$$

### 4.5. Optimization of Processes

The EE of EMO (Y_1_) and TET (Y_2_) were used as the index, with the overall score (OS) serving as the response value. The weights assigned to both were 0.5, and the OS was out of 100 points, calculated using the formula below (3). Based on previous experiments, three factors—lecithin dosage (A/mg), volume of phase B (B/mL), and stirring time (C/min)—and three levels were selected to optimize the process through the Box–Behnken response surface design method. The statistical analysis of the data was performed using Design-Expert 8.0.6.

(3) $$OS=Y_{1n}/Y_{1max}\times 0.5\times 100+Y_{2n}/Y_{2max}\times 0.5\times 100$$

### 4.6. Characterization of E-T/LNPs

The particle size and zeta potential of E-T/LNPs were determined by a particle analyzer (Litesizer 500, Anton Paar, Austria). The E-T/LNPs were dissolved in water, PBS, ethanol, and DMSO, respectively, followed by dissolving an equal volume of B/LNPs in water, and then an equal amount of EMO and TET in E-T/LNPs was dissolved in ethanol. All samples were diluted to the same concentration and sequentially exposed to visible light and fluorescence. The microscopic morphology of E-T/LNPs was observed by TEM. E-T/LNPs were stored at 25 °C room temperature, and their stability was investigated by measuring particle size, PDI, EE, and DL at days 0, 1, 10, and 30.

FT-IR: F127, EMO, TET, B/LNPs, E-T/LNPs, and the same ratio of EMO and TET mixture (E-T) were each mixed with KBr, pressed into pellets. FT-IR was scanned and analyzed at wavelengths between 4000 and 400 cm^−1^ to confirm the formation of E-T/LNPs.

Drug release in vitro: A phosphate buffer (with 0.78% tween-80, *v*/*v*) was used as dissolution medium for determination by dialysis [52]. Briefly, 1 mL of E-T/LNPs was sealed in a dialysis bag (3.5 kDA) and placed into 10 mL of the release medium. An equal amount of pure drug was taken, dissolved in 1 mL of water, and placed in the release medium under the same conditions as the control group. At 37 °C and 100 rpm, 2 mL of the release medium was sampled at 0.5, 1, 2, 4, 6, 8, 10, 12, and 24 h and replenished with an equal volume of fresh release medium at the same temperature. According to the method detailed in Section 4.4 and Equation (4), the CR was calculated.

(4) $${CR}_{n}\left(\%\right)=m_{1}+m_{2}{+m_{3}+\ldots +m}_{n}/The\;weight\;of\;drug\;added\times 100\%$$

Note: m_1_ is the mass of the drug measured in the first stage and so on.

### 4.7. Toxicity–Efficacy Concentrations of E-T/LNPs

AB line zebrafish larvae 48 h post-fertilization (hpf) were randomly selected (20 larvae per well) and given different doses of E-T/LNPs (0 μM, 2 μM, 3 μM, 4 μM, in terms of EMO content) for co-culture over 72 h. The zebrafish were then anesthetized with trichloroacetic acid after being freshened with the embryonic medium. Survival, healthy rate, and growth status were observed, photographed, and recorded by stereomicroscope (M165-FC, Lei-Ca, Germany).

(5) $$Survival\left(\%\right)=The\;number\;of\;surviving\;zebrafish/The\;total\;number\;of\;all\;zebrafish\times 100\%$$

(6) $$\begin{array}{c} Healthy\;rate\left(\%\right)=The\;number\;of\;zebrafish\;with\;normal\;morphology/The\;total\;number\;of\;all\;zebrafish\times 100\% \end{array}$$

### 4.8. Toxicity Evaluation of E-T/LNPs

Healthy AB larvae (20 larvae per well) were selected and divided into the Ctrl group, EMO group, E-T group, and E-T/LNPs group. The drugs for each group were diluted to the same concentration and then administered separately to each group (calculated based on the actual content of EMO in E-T/LNPs); the Ctrl group received blank culture water as a negative control. All groups were cultured for 72 h.

### 4.9. Anti-Liver Fibrosis In Vivo

Establishment and treatment of liver fibrosis in zebrafish: Zebrafish larvae at 2 dpf were divided into two groups: the Ctrl group and the LF Model group. The LF Model group was cultured in a medium incubated with 10 mM TAA to induce liver fibrosis. At 5 dpf, the LF Model group was further divided into five subgroups: Model, EMO (1.5 μM), TET (1.5 μM), E-T (1.5 μM), and E-T/LNPs (1.5 μM). The drugs were administered for 3 consecutive days, during which they were continuously cultured in TAA-containing medium. Observations were photographed at 8 dpf. The fluorescence area of their livers was quantified using Image J 1.54d [53].

Establishment and treatment of liver fibrosis in mice: Mice were divided into two groups. Mice in the LF Model group were injected intraperitoneally with corn oil (5 mL/kg, *v*/*v*) containing 20% CCl_4_ (three times a week) for 5 weeks to establish a model of liver fibrosis [54]. The Ctrl group was injected with equal volumes of saline. In the 6th week, the modeling mice were divided into the LF Model group, SIL group (positive drug silymarin), EMO group, E-T group, and E-T/LNPs group. Saline, SIL suspension (50 mg/kg) [55], EMO suspension (20 mg/kg) [9], EMO and TET suspension (20 mg/kg, in terms of EMO content), and E-T/LNPs solution (20 mg/kg, in terms of EMO content) were administrated orally, respectively. Treatment was given orally every 2 days for a total of 3 weeks, and corn oil with 20% CCl_4_ was administered continuously. The weight and growth of the mice were recorded during the experiment.

### 4.10. Sample Collection and Processing

At the end of the 8th week, the mice fasted for 12 h, the mice were euthanized by injection of pentobarbital sodium, and then their blood and liver were collected. A portion of liver tissue, fixed in paraformaldehyde solution for 48 h, was paraffin-embedded and used for HE and Masson staining. The rest of the liver tissue was processed according to the instructions of the ELISA kit to obtain liver homogenate supernatant. All samples were stored at −80 °C for later experiments.

### 4.11. Biochemical Index Detection

Mice serum samples were used to detect liver function (AST, ALT), and mice liver homogenates were used to detect markers of liver fibrosis (PC III, Col IV, LN, HA). All procedures were carried out according to the instructions of the kit.

### 4.12. Histopathological

Liver tissues were sectioned and stained with HE, and images were acquired at 100× and 400× magnifications, respectively, using a digital sectioning scanner (Pannoramic 250, 3DHISTECH, Budapest, Hungary). The tissues were also stained using Masson trichrome staining, based on the principle that collagen fibers appear blue and muscle fibers red. Images were captured at 400× magnification by a digital trinocular camera microscope (BA210Digital, Motic Industrial Group Ltd., Xiamen, China). Finally, the blue area was quantified using Image J 1.54d.

### 4.13. Immunohistochemistry

The fixed sectioned liver tissue was deparaffinized and rehydrated. After overnight incubation with antibodies at 4 °C under dark conditions, specific antibodies were added for co-incubation. Finally, the shots were observed under a microscope and the expressions of IL-1β and TGF-β were quantified by Image J 1.54d.

### 4.14. Data Analysis

All data were compiled and analyzed through IBM SPSS 26.0. One-way analysis of variance (ANOVA) was used for statistical data, and mean and standard deviation were used for quantitative data. The significance of all results was judged by *p* < 0.05. Plots were performed using Origin 2023b.

## 5. Conclusions

In this study, the LNPs containing EMO and TET were successfully prepared, which effectively reduced the toxicity of EMO and increased the toxicity–efficacy concentration of E-T/LNPs in vivo. At low doses, E-T/LNPs can treat hepatic fibrosis, while at high doses, they may cause toxicity. Liver fibrosis is an important stage in the transition from hepatitis to cirrhosis and hepatocellular carcinoma. E-T/LNPs can treat liver fibrosis and restore the normal physiological function of the liver, and bring hope for the treatment of chronic liver diseases. In summary, this novel combination of EMO and TET may be one of the future ways to treat liver fibrosis.

## Data Availability

The data presented in this study are available upon request from the corresponding author.

## Supplementary Materials

The following supporting information can be downloaded at: https://www.mdpi.com/article/10.3390/molecules29215122/s1, Table S1: Factors and levels of Box-Behnken response surface design; Table S2: ANOVA results; Table S3: Box-Behnken response surface results; Figure S1: 3D diagram and contours of the Box-Behnken response surface; Table S4: Particle size, PDI, EE, and DL of E-T/LNPs; Figure S2: Cumulative release in PBS, pure EMO and TET demonstrated significant differences in the monomer drug group and E-T/LNPs group.
