# Supplementary material for: Study on the Hepatotoxicity of Emodin and Its Application in the Treatment of Liver Fibrosis
Source: Molecules. 2024 Oct 30;29(21):5122. doi: 10.3390/molecules29215122 (PMC11547690; doi:10.3390/molecules29215122)
Supplement: Supplementary file 1 [file molecules-29-05122-s001.zip › molecules-3186010-supplementary.pdf]

Table S1 Factors and levels of Box-Behnken response surface design

Level	A/mg	B/mL	C/min
1	600	20	60
0	500	15	50
-1	400	10	40

Table S2 ANOVA results

Source	Sum of Squares	df	Mean Square	F Value	p-value
Model	1352.19	9	150.24	5.06	0.0220
A	15.18855	1	15.19	0.51	0.4977
B	318.7419	1	318.74	10.73	0.0136
C	163.0692	1	163.07	5.49	0.0516
AB	33.45659	1	33.46	1.13	0.3238
AC	0.286223	1	0.29	0.01	0.9246
BC	38.84713	1	38.85	1.31	0.2904
A2	18.00014	1	18.00	0.61	0.4618
B2	552.4926	1	552.49	18.60	0.0035
C2	157.73	1	157.73	5.31	0.0547
Residual	207.9531	7	29.71		
Lack of Fit	71.02431	3	23.67	0.69	0.6033
Pure Error	136.9288	4	34.23		
Cor Total	1560.143	16			

Table S3 Box-Behnken response surface results

NO	A	B	C	OS
1	0	-1	1	52.34
2	0	0	0	91.49
3	0	1	1	73.24
4	-1	1	0	76.28
5	0	-1	-1	72.15
6	1	0	1	79.47
7	1	1	0	81.56
8	1	0	-1	84.49
9	1	-1	0	65.19
10	0	0	0	83.55
11	0	0	0	92.47
12	0	0	0	89.55
13	0	1	-1	80.58
14	-1	-1	0	71.49
15	-1	0	1	73.98
16	-1	0	-1	77.93
17	0	0	0	78.71

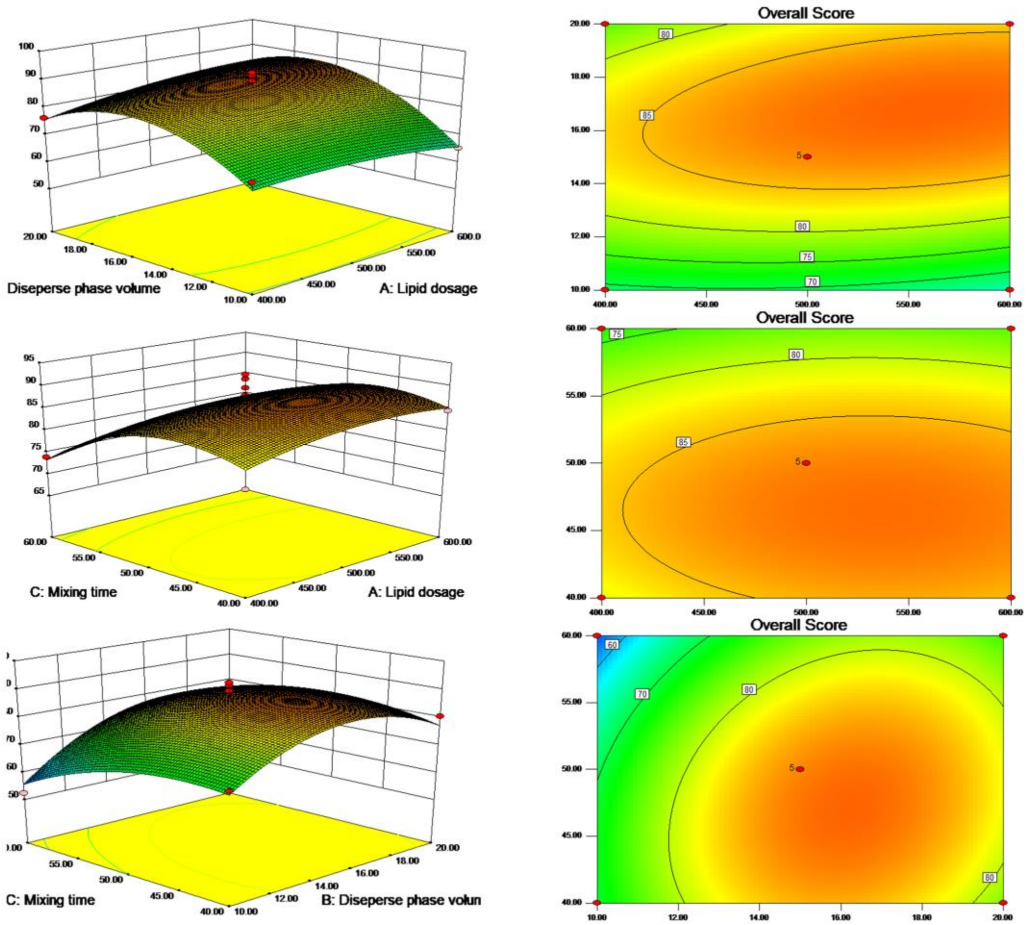

Figure S1 3D diagram and contours of the Box-Behnken response surface

Table S4 Particle size, PDI, EE, and DL of E-T/LNPs

Days	Size(nm)	PDI(%)	EE(%)		DL(%)	
			EMO	TET	EMO	TET
0	73.12±0.88	17.4±5.3	90.65	98.82	4.87	5.28
1	73.30±0.50	17.5±2.4	90.99	94.66	4.88	5.07
10	77.80±1.01	18.2±7.0	87.93	78.08	4.73	4.22
30	81.07±1.44	17.8±3.4	73.54	75.08	3.99	4.07

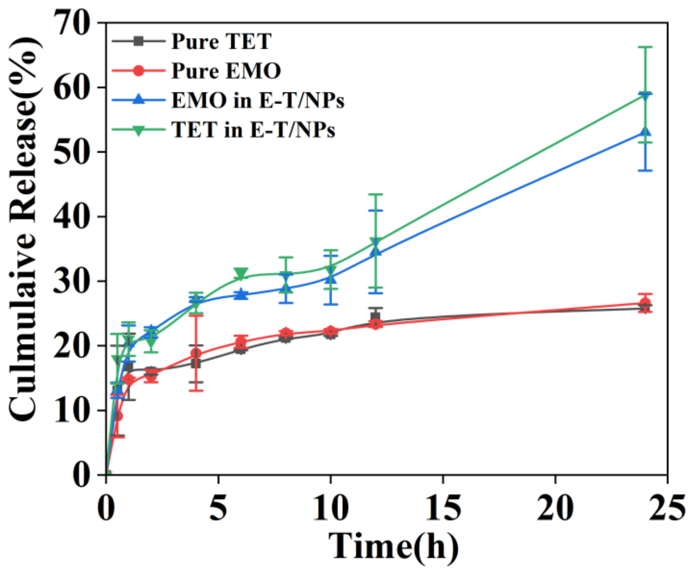

Figure S2 Cumulative release in PBS, pure EMO and TET demonstrated significant differences in the monomer drug group and E-T/LNPs group
